# TBX18 overexpression enhances pacemaker function in a rat subsidiary atrial pacemaker model of sick sinus syndrome

**DOI:** 10.1113/JP276508

**Published:** 2018-10-13

**Authors:** M. Choudhury, N. Black, A. Alghamdi, A. D'Souza, R. Wang, J. Yanni, H. Dobrzynski, P. A. Kingston, H. Zhang, M. R. Boyett, G. M. Morris

**Affiliations:** ^1^ Institute of Cardiovascular Sciences University of Manchester Manchester UK

**Keywords:** biopacemaking, gene therapy, sick sinus syndrome, subsidiary atrial pacemaker tissue, TBX18

## Abstract

**Key points:**

- The sinoatrial node (SAN) is the primary pacemaker of the heart. SAN dysfunction, or ‘sick sinus syndrome’, can cause excessively slow heart rates and pauses, leading to exercise limitation and syncope, currently treated by implantation of an electronic pacemaker.
- ‘Biopacemaking’ utilises gene therapy to restore pacemaker activity by manipulating gene expression. Overexpressing the HCN pacemaker ion channel has been widely used with limited success.
- We utilised bradycardic rat subsidiary atrial pacemaker tissue to evaluate alternative gene targets: the Na^+^/Ca^2+^ exchanger NCX1, and the transcription factors TBX3 and TBX18 known to be involved in SAN embryonic development.
- TBX18 overexpression restored normal SAN function, as assessed by increased rate, improved heart rate stability and restoration of isoprenaline response. TBX3 and NCX1 were not effective in accelerating the rate of subsidiary atrial pacemaker tissue.
- Gene therapy targeting TBX18 could therefore have the potential to restore pacemaker function in human sick sinus syndrome obviating electronic pacemakers.

**Abstract:**

The sinoatrial node (SAN) is the primary pacemaker of the heart. Disease of the SAN, sick sinus syndrome, causes heart rate instability in the form of bradycardia and pauses, leading to exercise limitation and syncope. Biopacemaking aims to restore pacemaker activity by manipulating gene expression, and approaches utilising HCN channel overexpression have been widely used. We evaluated alternative gene targets for biopacemaking to restore normal SAN pacemaker physiology within bradycardic subsidiary atrial pacemaker (SAP) tissue, using the Na^+^/Ca^2+^ exchanger NCX1, and the transcription factors TBX3 and TBX18. TBX18 expression in SAP tissue restored normal SAN function, as assessed by increased rate (SAN 267.5 ± 13.6 bpm, SAP 144.1 ± 8.6 bpm, SAP‐TBX18 214.4 ± 14.4 bpm; *P* < 0.001), improved heart rate stability (standard deviation of RR intervals fell from 39.3 ± 7.2 ms to 6.9 ± 0.8 ms, *P* < 0.01; root mean square of successive differences of RR intervals fell from 41.7 ± 8.2 ms to 6.1 ± 1.2 ms, *P* < 0.01; standard deviation of points perpendicular to the line of identity of Poincaré plots (SD1) fell from 29.5 ± 5.8 ms to 7.9 ± 2.0 ms, *P* < 0.05) and restoration of isoprenaline response (increases in rates of SAN 65.5 ± 1.3%, SAP 28.4 ± 3.4% and SAP‐TBX18 103.3 ± 10.2%; *P* < 0.001). These changes were driven by a TBX18‐induced switch in the dominant HCN isoform in SAP tissue, with a significant upregulation of HCN2 (from 1.01 × 10^−5^ ± 2.2 × 10^−6^ to 2.8 × 10^−5^ ± 4.3 × 10^−6^ arbitrary units, *P* < 0.001). Biophysically detailed computer modelling incorporating isoform‐specific HCN channel electrophysiology confirmed that the measured changes in HCN abundance could account for the observed changes in beating rates. TBX3 and NCX1 were not effective in accelerating the rate of SAP tissue.

## Key points

- The sinoatrial node (SAN) is the primary pacemaker of the heart. SAN dysfunction, or ‘sick sinus syndrome’, can cause excessively slow heart rates and pauses, leading to exercise limitation and syncope, currently treated by implantation of an electronic pacemaker.
- ‘Biopacemaking’ utilises gene therapy to restore pacemaker activity by manipulating gene expression. Overexpressing the HCN pacemaker ion channel has been widely used with limited success.
- We utilised bradycardic rat subsidiary atrial pacemaker tissue to evaluate alternative gene targets: the Na^+^/Ca^2+^ exchanger NCX1, and the transcription factors TBX3 and TBX18 known to be involved in SAN embryonic development.
- TBX18 overexpression restored normal SAN function, as assessed by increased rate, improved heart rate stability and restoration of isoprenaline response. TBX3 and NCX1 were not effective in accelerating the rate of subsidiary atrial pacemaker tissue.
- Gene therapy targeting TBX18 could therefore have the potential to restore pacemaker function in human sick sinus syndrome obviating electronic pacemakers.

## Introduction

The heart's natural pacemaker, the sinoatrial node (SAN), is a complex and heterogeneous tissue capable of reliably initiating each heartbeat in a robust manner under varying physiological conditions (Monfredi *et al*. 2010). Sinus node disease, or sick sinus syndrome (SSS), is the commonest bradyarrhythmia in humans, and will be of increasing importance with an ageing population (Jensen *et al*. 2014). The clinical spectrum includes periodic sinus bradycardia, chronotropic incompetence and paroxysmal sinus pauses. Thus the disease may present as exercise limitation or syncope and is the leading indication for electronic pacemaker implantation (Mond & Proclemer, 2011). Electronic pacemakers palliate the symptoms of SSS but have known limitations including the lack of responsiveness to autonomic modulation, the requirement for frequent battery changes, and up to 12.4% implant complication rate (Brignole *et al*. 2013). Biopacemaking attempts to recreate pacemaker tissue similar to the SAN elsewhere in the heart by manipulating pacemaker genes to create a safer, more physiological and curative treatment for bradyarrhythmia (Rosen *et al*. 2011).

The effect of HCN channel over‐expression in myocardium has been widely studied as a biopacemaker strategy with some success (Rosen *et al*. 2011). However, HCN channels display ‘context dependence’ and so may function differently when expressed in ectopic (i.e. non‐SAN) tissue and this may account for some of the difficulties in obtaining a physiologically appropriate heart rate that have been encountered with this approach (Siu *et al*. 2006; Plotnikov *et al*. 2008). Robust SAN pacemaking relies on the interaction of several mechanisms including mutually entrained membrane and Ca^2+^ ‘clocks’, of which NCX is an important component, as well as complexity in SAN ultrastructure (Monfredi *et al*. 2010). Therefore the human embryonic transcription factors T‐box 3 (TBX3) and T‐box 18 (TBX18) are attractive targets for biopacemaking as they are important in SAN development and so have the potential to have a broad effect on cardiac tissue phenotype (Wiese *et al*. 2009). Ectopic expression of TBX18 in ventricular cells has been shown to reprogram ventricular cardiomyocytes into SAN‐like cells that can reduce the need for electronic ventricular pacing in animal models of atrioventricular block (Kapoor *et al*. 2013; Hu *et al*. 2014). However, patients with SSS require atrial or dual chamber pacing to maintain cardiac synchrony and prevent ‘pacemaker syndrome’ (Sweeney *et al*. 2003; Link *et al*. 2004). We have previously demonstrated that expression of a chimaeric HCN channel can accelerate spontaneous pacing in bradycardic subsidiary atrial pacemaker (SAP) tissue suggesting its utility in SSS (Morris *et al*. 2013), but biopacemaker strategies for SSS employing targets other than HCN genes have not been studied.

In these experiments TBX18 expression was targeted to the inferior extension of the crista terminalis (CT). This area was chosen because the tissue contains cells with a nodal phenotype (Morris *et al*. 2013). For example, when compared to working atrial myocytes the cells in this area are smaller, express relatively high levels of HCN channels, and there are high levels of collagen, much like the central SAN (Morris *et al*. 2013). Furthermore this area is a functional extension of the SAN as part of an extended SAN complex (Boineau *et al*. 1988; Stiles *et al*. 2010; Morris & Kalman, 2014). This region becomes an important pacemaker zone in SSS when the primary pacemaker moves away from the dysfunctional central SAN (Joung *et al*. 2010, 2011; Yanni *et al*. 2010). The tissue here displays spontaneous CsCl‐sensitive (i.e. *I*_f_ dependent) pacemaker activity, with action potentials similar to the SAN (e.g. diastolic depolarisation slope, low maximum diastolic potential, slow maximum depolarisation rate) but is bradycardic with a high degree of overdrive suppression and low catecholamine sensitivity (Rozanski *et al*. 1984; Rubenstein *et al*. 1987; Rubenstein & Lipsius, 1989).

This present study was designed to assess the effectiveness of alternative non‐HCN gene targets for biopacemaker treatment of SSS (NCX, TBX3 and TBX18), and then, for the gene target meeting the primary target of rate acceleration, to characterise biopacemaker physiology in further detail to inform the potential to treat the clinical spectrum of heart pacemaker dysfunction in human SSS which goes beyond simple bradycardia.

## Methods

### Ethical approval

Two‐month‐old male Wistar–Hannover rats weighing 300–350 g were used for this study. They had free access to food and water. Animals were euthanised humanely by a Schedule 1 procedure (concussion and cervical dislocation) in accordance with Home Office regulations under the Animals (Scientific Procedures) Act 1986 under an institutional licence held by the University of Manchester. Anaesthesia was not required and no other procedures were performed.

### Tissue culture and characterisation of spontaneous pacing behaviour

Isolation of the SAN and the method of preparation of the SAP tissue have been previously described in detail (Morris *et al*. 2013). The SAN preparation was isolated by dissecting the entire right atrium (RA) in sterile Tyrode solution at 37°C; this was then opened along the anterior atrial wall and anterior superior vena cava so that the posterior intercaval region remained intact. The SAP tissue was isolated by dividing the RA preparation horizontally at the level of the fossa ovalis removing the superior section along with removal of the atrioventricular node as previously described (Morris *et al*. 2013). The tissue was cultured in Advanced Dulbecco's Modified Eagle's Medium/Ham's F‐12 (DMEM/F12, Thermo Fisher Scientific, Waltham, MA, USA) supplemented with 10% fetal bovine serum (Thermo Fisher Scientific), and penicillin, 500 units ml^−1^ (Sigma‐Aldrich, St Louis, MO, USA); streptomycin, 0.5 mg ml^−1^ (Sigma‐Aldrich); l‐glutamine, 2 mM (Sigma‐Aldrich)) in a customised constant circulating superfusion system that maintained the sterile solution at 37°C and pH 7.4. Bipolar extracellular potentials were recorded using two 0.22 mm stainless steel electrodes and a Powerlab filter and amplifier system with Labchart software (ADInstruments, Sydney, Australia) at a sample rate of 10 kHz.

To assess the effect of the target gene on pacing rate, the average spontaneous pacing rate between 46 and 48 h of culture was measured. The first 12 h (i.e. before predicted significant transgene expression) was used as a control to ensure that the SAP preparations had comparable initial rates. Clinical SSS is associated with rate instability in the form of paroxysmal bradycardia and sinus pauses. To quantify pacemaker stability during this same 46–48 h period in culture, we measured the standard deviation of RR intervals (SDRR) and root mean square of successive differences of RR intervals (RMSDD), and generated a Poincaré plot (RR[*n*] *vs*. RR[*n*+1]) for each tissue preparation. The SD1 of the Poincaré plots were calculated (standard deviation of points perpendicular to the line of identity of the Poincaré plot) and plots were categorised into those with and without ectopic activity based on visual inspection.

Change in rate was assessed in the presence of β‐adrenergic stimulation by isoprenaline (ISO) 0.05 μM (Sigma‐Aldrich) and *I*_f_ blockade by CsCl 2 mM (Sigma‐Aldrich). In a separate set of experiments overdrive pacing was delivered via a coaxial stimulator electrode (Harvard Apparatus, Holliston, MA, USA) as 3 × 2 min pacing trains delivered at 75% of the spontaneous cycle length for each preparation. Pacing pulse width was 2 ms with voltage amplitude of 1.5× capture threshold. The corrected recovery time (cRT) was calculated as the difference between the recovery time (the time from the last paced beat to the next spontaneous beat) and the spontaneous cycle length. The untreated SAP tissue was used for the control rate as we have previously demonstrated that adenovirus carrying non‐functional ion channel protein or green fluorescent protein (GFP) does not affect the beating rate of the same SAP tissue preparation (Morris *et al*. 2013).

### Recombinant adenoviruses

Recombinant adenoviruses Ad‐TBX18, Ad‐TBX3 (ABMGood) and Ad‐NCX1‐GFP (kindly provided by Prof. Godfrey Smith) were amplified using a filter‐based AdEasy Virus Purification kit (Agilent Technologies, Santa Clara, CA, USA). A graduated syringe (Nanofil, WPI, Sarasota, FL, USA) was used to inject 1–2 μl of pre‐warmed (37°C) adenovirus via a 35G needle into the superior cut edge of the CT in the SAP preparations, to deliver ∼1 × 10^7^ pfu as previously described (Morris *et al*. 2013).

### PCR

Biopsy samples of 2 mm were taken and snap frozen after 48 h of tissue culture from (1) SAN preparations, at the area of the SAN; (2) the SAP preparations, at the inferior CT target for injection; (3) the adenovirus‐injected SAP preparations, at the site of injection. RNA was extracted using the MirVana kit (Thermo Fisher Scientific) and treated with Turbo DNase (Thermo Fisher Scientific). Total RNA was reverse transcribed to cDNA using the SuperScript VILO Master Mix kit (Thermo Fisher Scientific). RNA concentration was measured using a Nanodrop spectrophotometer. qPCR reactions were performed in 96‐well plates using SYBR Green fluorescent probe assays and each reaction was performed in triplicate. Transcript expression levels were calculated using the Δ*C*_t_ method; data in graphs represent the Δ*C*_t_ values. The ratio of mRNA abundance between the gene of interest and a housekeeping gene, *18S*, was calculated for each reaction, given as: (reaction efficiency)*^C^* ^t^ for *18S*/(reaction efficiency)*^C^* ^t^ for gene of interest.

### Modelling

To simulate the effect of HCN remodelling on SAN electrical activity, a mouse SAN mathematical model was used (Kharche *et al*. 2011). In the model *I*_f_ is represented as single current. The model was adapted to include an isoform‐specific *I*_f_, consisting of *I*_f,HCN_ *_1_*, *I*_f,HCN2_ and *I*_f,HCN4_, each of which considered the channel permeability to Na^+^ and K^+^ ions. Thus, the newly developed *I*_f_ is given by:

If=If, HCN 1+If, HCN 2+If, HCN 4

If, HCN 1=I fNa , HCN 1+I fK , HCN 1

If, HCN 2=I fNa , HCN 2+I fK , HCN 2

If, HCN 4=I fNa , HCN 4+I fK , HCN 4

Experimental characteristics of HCN channels were used (Zong *et al*. 2012) to determine the activation midpoint for the three HCN isoforms and were applied in the mouse SAN model as: *V*_0.5_ = −75.2 mV for *I*_f,HCN1_, −92.0 mV for *I*_f,HCN2_ and −91.2 mV for *I*_f,HCN4_. The activation time constants of the individual HCN1, HCN2 and HCN4 isoforms were also simulated in this model according to the experimental measurements (Herrmann *et al*. 2007).

The relative contributions of HCN1, HCN2 and HCN4 to whole‐cell *I*_f_ were also considered in the model. The maximum ionic current conductance was assigned proportionally to the isoforms: HCN4 was considered to be the largest component of the native *I*_f_ and the ratio among these three isoforms (*g*_f,HCN1_:*g*_f,HCN2_:*g*_f,HCN4_) was 3:1:6. Model equations and parameters for *I*_f_ were validated by quantitatively comparing the simulated *I–V* relationship with experimental data (Herrmann *et al*. 2007; Baruscotti *et al*. 2011; El Khoury *et al*. 2013).

The purpose of the simulation was to investigate the predicted changes to *I*_f_ and the pacemaker rate in each preparation (SAN, SAP and SAP‐TBX18) based on the measured HCN isoform mRNA abundances. The relative contribution of each HCN isoform to total *I*_f_ was adjusted according to the measured mRNA level for each preparation assuming that the mRNA level is representative of functional channel protein expression level at the cell membrane (Fenske *et al*. 2013). This method does not aim to generate definitive biophysically detailed action potential models, but is a form of bioinformatics to explore the possible consequences of changes in transcript levels.

### Statistics

Data are presented as the mean ± SEM, and statistical significance was tested using multiple ANOVA or chi‐square test for categorical data.

## Results

### Effect of transgene expression on pacemaker activity: spontaneous pacemaker rate

**Figure 1:**
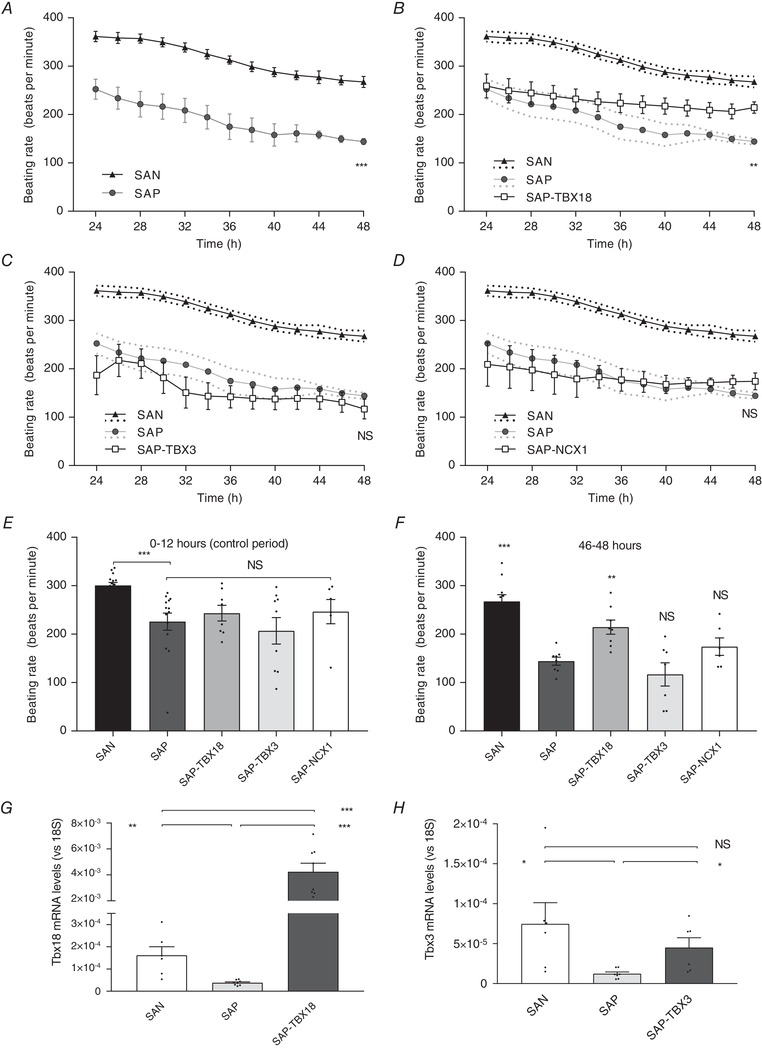
Adenovirus‐mediated expression of TBX18, but not NCX or TBX3, increases the beating rate of the SAP preparation *A*, uninfected SAP beating rates were significantly slower than the SAN. *B*, in SAP‐TBX18 beating rates diverged from uninfected SAP tissue after 20 h of culture, and were significantly faster than SAP at the final analysis (*F*). The rates during the control period were not significantly different (*E*). *C* and *D*, there was no significant effect on the rate when the SAP preparation was injected with Ad‐TBX3 or Ad‐NCX1‐GFP. Statistical significance displayed in panels *A–D* refers to the pre‐specified comparison of the final 2 h rates between treated and untreated SAP by multiple ANOVA. Statistical significance in *E* and *F* refers to comparison made to the rate of the untreated SAP preparation. *G* and *H*, expression of TBX18 and TBX3 was validated by qPCR, showing significant upregulation in treated *versus* untreated SAP preparations. Comparisons were also made to SAN preparations. SAN, *n* = 15; SAP, *n* = 14; SAP‐TBX18, *n* = 8; SAP‐TBX3, *n* = 9; SAP‐NCX1, *n* = 6. NS, not significant; ^**^ *P* < 0.01; ^***^ *P* < 0.001. Dotted lines in *B–D* represent SEM. Individual data points are shown by dots in bar graphs.

Tissue preparations were maintained in culture for 48 h and a pre‐specified beating rate analysis was taken between 46 and 48 h (Fig. 1). Direct expression of transgenes may be expected to have a physiological effect on pacing by 24 h (Morris *et al*. 2013), whereas the effect of transcription factors such as TBX3 or TBX18 is predicted to take a minimum of 36 h (Kapoor *et al*. 2013). During the final 2 h of culture, the uninjected SAP preparations (*n* = 14) were relatively bradycardic; the rate was significantly slower than that of the SAN preparations (*n* = 15) (144.1 ± 8.6 *vs*. 267.5 ± 13.6 bpm, respectively, *P* < 0.01). The SAN rate was significantly faster at all time points when compared to SAP (Fig. 1 *A*). SAP preparations were injected with a recombinant adenovirus into a region near the inferior projection of the CT previously demonstrated to contain bradycardic pacemaker tissue (Morris *et al*. 2013). In SAP‐TBX18 (*n* = 8, Fig. 1 *B*) there was a trend for beating rates to diverge from uninfected SAP tissue after 20 h of culture, becoming statistically significant by 44 h. There was no significant effect on the rate when the SAP preparation was injected with Ad‐TBX3 or Ad‐NCX1‐GFP (Fig. 1 *C* and *D*; not significant (NS) compared to untreated SAP). The rates during the control period were not significantly different (SAP‐TBX18 247.8 ± 15.7 bpm *vs*. SAP 225.2 ± 19.4 bpm, NS, Fig. 1 *E*). Out of all treated SAP preparations at the final analysis (between 46 and 48 h) only SAP‐TBX18 was significantly faster than untreated SAP tissue (214.4 ± 14.4 *vs*. 144.1 ± 8.6 bpm, *P* < 0.01, Fig. 1 *F*). We therefore selected SAP‐TBX18 for further detailed characterisation.

Expression of TBX18 and TXB3 was validated by qPCR with a significant rise in mRNA abundance of both gene targets when comparing untreated and treated preparations (Fig. 1 *G* and *H*). Expression of NCX1 was validated visually on confocal microscopy via bicistronic expression of GFP protein with strong signal seen only in treated preparations (data not shown).

### Effect of transgene expression on pacemaker activity: pacemaker stability

**Figure 2:**
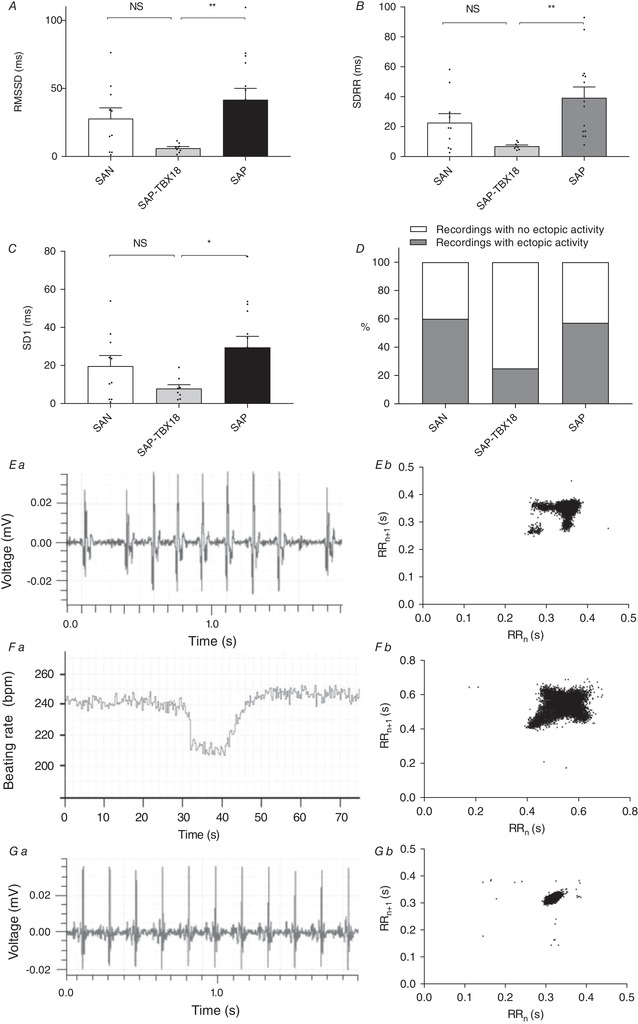
TBX18 improves heart rate stability in the SAP *A–C*, compared to untreated SAP there was a significant improvement in all measures of heart rate stability studied in SAP‐TBX18. *D*, Poincaré plots displayed fewer clusters of outlying short‐coupled RR intervals or pauses in SAP‐TBX18 preparations, though this did not reach significance. These premature beats (such as the example shown in *Eb*) demonstrated a change in morphology suggesting an ectopic focus of origin. *E*, examples of heart rate behaviour in the SAN; *Ea*, example extracellular potential recording from SAN preparation that showed rate instability with sudden changes in RR intervals; *Eb*, example Poincaré plot from a SAN preparation that displayed heart rate instability with high SD1 and outlying clusters representing short RR intervals and pauses. *F*, examples of unstable heart rate behaviour in SAP tissue; *Fa*, example RR plot from a SAP preparation that showed rate instability with sudden heart rate decelerations; *Fb*, example Poincaré plot from a SAP preparation that displayed heart rate instability with high SD1 but without outlying clusters. *G*, examples of stable heart rate behaviour in SAP‐TBX18 preparations; *Ga*, example extracellular potential recording from a SAP‐TBX18 preparation that had a stable rate and minimal variation in RR intervals; *Gb*, example Poincaré plot from SAP‐TBX18 preparation that displayed a stable heart rate with low SD1 and no outlying clusters. SAN, *n* = 10; SAP, *n* = 14; SAP‐TBX18, *n* = 8. NS, not significant; ^*^ *P* < 0.05; ^**^ *P* < 0.01. Individual data points are shown by dots in bar graphs.

In addition to bradycardia, a feature of SSS is rate instability in the form of pauses and atrial arrhythmia (Morris & Kalman, 2014). We analysed pacing behaviour from 46 to 48 h using parameters of heart rate variability (HRV) to quantify rate stability in SAN (*n* = 10), SAP (*n* = 14) and SAP‐TBX18 (*n* = 8); for these *ex vivo* experiments, in the absence of autonomic input, low HRV will indicate stable pacemaker activity. Compared to untreated SAP, SAP‐TBX18 displayed significantly improved parameters of rate stability; SDRR reduced from 39.3 ± 7.2 to 6.9 ± 0.8 ms (*P* < 0.01, Fig. 2 *A*), RMSSD reduced from 41.7 ± 8.2 to 6.1 ± 1.2 ms (*P* < 0.01, Fig. 2 *B*) and the SD1 of the Poincaré plot reduced from 29.5 ± 5.8 to 7.9 ± 2.0 ms (*P* < 0.01, Fig. 2 *C*). Pauses and premature short RR intervals are revealed as outlying clusters on Poincaré plots, seen in 25% of SAP‐TBX18 and 57% of untreated SAP (Fig. 2 *D*, NS by chi‐square test). Short RR coupling may represent ectopic beats or early primary pacemaker firing; it was not possible to perform activation mapping to confirm the origin of these beats, but they often demonstrated a change in morphology as seen in the raw signal data (example from the SAN shown in Fig. 2 *Ea*), showing a different amplitude or axis, meaning they were likely to be originating from an alternative focus. Representative traces of stable and unstable rate behaviour are shown in Fig. 2 *E–G* along with representative Poincaré plots.

### Effect of transgene expression on pacemaker activity: pacemaker physiology

For each preparation we assessed the β‐adrenergic responsiveness using isoprenaline (Iso). The SAP model had reduced Iso sensitivity in comparison to SAN (acceleration in rate of 27.8 ± 2.9% *vs*. 69.5 ± 12.6%, respectively, *P* < 0.05). In SAP‐TBX18 the Iso response was restored (acceleration in rate of 121.3 ± 4.2% *vs*. SAP 27.8 ± 2.9%, *P* < 0.001). The normal SAN has very little overdrive suppression in response to rapid pacing, whereas subsidiary pacemaker tissues such as Purkinje fibres show prolonged suppression of pacemaker activity after periods of overdrive pacing, as does the diseased SAN (Boyett & Fedida, 1984; Zipes *et al*. 1989). We assessed the response of the tissue preparations to overdrive pacing. The cRT of the SAP was significantly longer than the SAN (124.6 ± 10.1 *vs*. 77.3 ± 9.5 ms, respectively, *P* < 0.01). TBX18 did not significantly shorten the cRT when compared to untreated SAP (140.0 ± 23.1 *vs*. 124.6 ± 10.1 ms, respectively, NS).

### The effect of TBX18 on gene expression in the subsidiary pacemaker region

**Figure 3:**
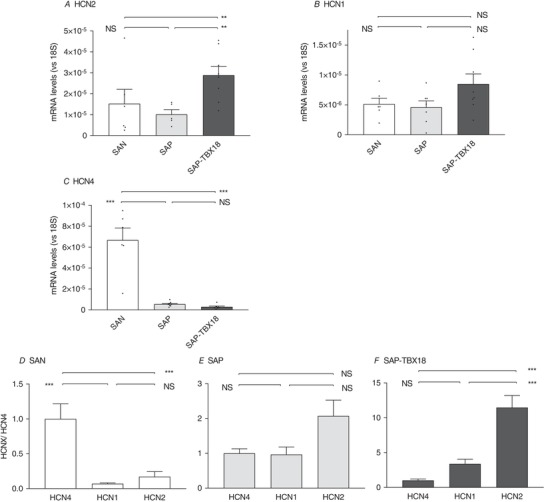
Comparison of mRNA levels of HCN isoforms *A–C*, relative abundance of HCN transcripts as determined by qPCR in the SAN, SAP and SAP‐TBX18. *D–F*, ratios of HCN isoforms in SAN, SAP and SAP‐TBX18. Levels are shown relative to HCN4, which is the most abundant isoform in the SAN. TBX18 causes a change in the relative levels of the isoforms in the SAP preparation with a significant upregulation of HCN2 (*F*) compared to the untreated SAP (*E*). SAN, *n* = 8; SAP, *n* = 8; SAP‐TBX18, *n* = 8. NS, not significant; ^*^ *P* < 0.05; ^**^ *P* < 0.01; ^***^ *P* < 0.001. Individual data points are shown by dots in bar graphs.

mRNA levels were assessed after 48 h culture using RT‐qPCR (*n* = 8 replicates per condition). Due to the positive chronotropic effect of TBX18 on the pacemaker activity of SAP tissue as demonstrated above, HCN channel expression was compared to the SAN. HCN channels are responsible for the hyperpolarization‐activated current, *I*_f_, an important pacemaker current (Monfredi *et al*. 2010). In the TBX18‐treated SAP tissue there was a switch in the dominant HCN isoform with a significant upregulation of HCN2 from 1.01 × 10^−5^ ± 2.2 × 10^−6^ to 2.8 × 10^−5^ ± 4.3 × 10^−6^ arbitrary units (Fig. 3 *A*, *P* < 0.01) and a trend for an upregulation in HCN1 which did not reach significance (Fig. 3 *B*, *P* = 0.076). HCN4 was the most abundant isoform in the SAN (Fig. 3 *C*, *P* < 0.001). Using the abundance of HCN4 as the reference, the ratios of the HCN isoforms (HCN4/HCN1/HCN2, respectively) were; SAN, 1/0.07/0.17; SAP, 1/0.97/2.07; SAP‐TBX18, 1/3.3/11.5 (Fig. 3 *D–F*, *P* < 0.001 for ratios of isoform expression across the three tissue groups by chi‐squared).

**Figure 4:**
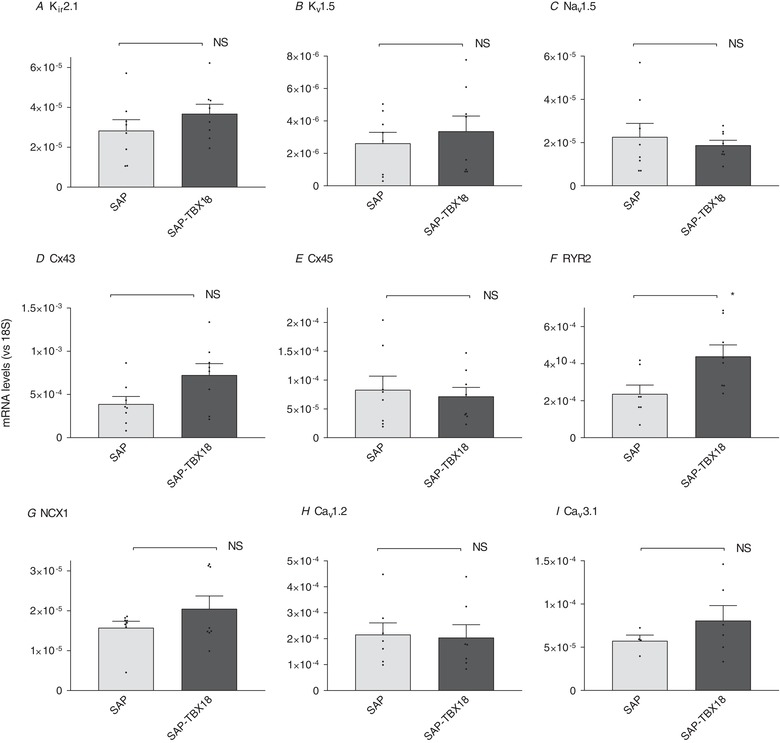
Relative abundance of further ion channels relevant to the normal sinus node as determined by qPCR in SAP and SAP‐TBX18 Only RYR2 demonstrated a significant rise in SAP‐TBX18 compared to untreated SAP. There were no significant changes in the other measured genes. SAN, *n* = 8; SAP, *n* = 8; SAP‐TBX18, *n* = 8. NS, not significant; ^*^ *P* < 0.05. Individual data points are shown by dots in bar graphs.

We also assessed the effect of TBX18 expression on transcripts in the SAP known to be important for normal SAN function. The ryanodine receptor RYR2 makes an important contribution to pacemaking via the SAN Ca^2+^ clock (Monfredi *et al*. 2010). TBX18 caused a significant up‐regulation of RYR2, but other components of the SAN pacemaker clock were not changed and levels of the connexins Cx43 and Cx45 were not altered by TBX18 expression (Fig. 4).

### Detailed computer modelling

**Figure 5:**
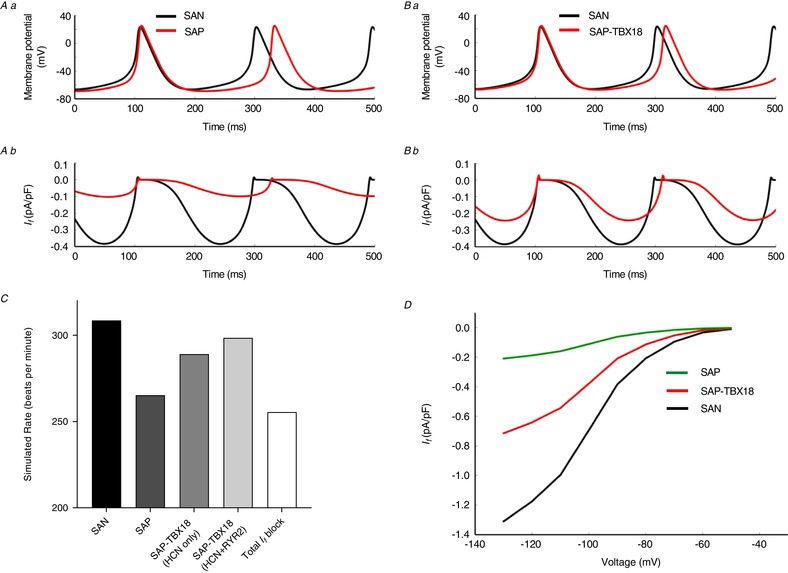
Biophysically detailed computer modelling based on measured HCN mRNA abundances *A* and *B*, computed membrane potentials (*Aa* and *Ba*) and *I*_f_ (*Ab* and *Bb*) are shown. The SAN (black lines) was compared to SAP (red lines, *Aa* and *Ab*) and to SAP‐TBX18 (red lines *Ba* and *Bb*). *C*, simulated heart rates; SAP‐TBX18 (HCN only) refers to the simulation only accounting for changes to HCN channel levels; SAP‐TBX18 (HCN+RYR2) refers to the simulation accounting for changes to HCN channel levels and RYR2 levels. *D*, simulated current–voltage relationships for each preparation normalised to cell capacitance. [Color figure can be viewed at wileyonlinelibrary.com]

We used a detailed computer model of the SAN to test the ability of the measured HCN mRNA abundances to account for the observed changes in beating rates assuming that the changes in mRNA resulted in corresponding changes in protein and *I*_f_. The simulation results confirmed that the improvement in pacemaker rate of SAP‐TBX18 is primarily explained by the observed upregulation of HCN channels and consequent increase in *I*_f_. SAN action potentials were simulated according to the measured HCN mRNA abundances and are shown in Fig. 5. In the case of SAP the simulated *I*_f_ density decreased and the diastolic depolarisation slope (DD) was reduced from 0.23 V s^−1^ to 0.17 V s^−1^; there was prolongation of the computed cycle length manifesting as a reduction in rate from 308.6 to 265.3 bpm (Fig. 5 *A–D*). The simulated action potentials for SAP‐TBX18 are shown in Fig. 5 *B*. In comparison to the SAN, the simulated *I*_f_ density was decreased and DD was slower at 0.2 V s^−1^ (Fig. 5 *D*) and there was an increase in the computed cycle length, manifesting as a reduction in rate from 308.6 to 288.8 bpm (Fig. 5 *B* and *C*). Further adjustment of the simulation to account for the increased RYR2 mRNA abundance observed in SAP‐TBX18 in addition to the increase in HCN abundances led to a small increase in the simulated beating rate to 298.5 bpm (Fig. 5 *C*). These data are qualitatively consistent with the observed differences in beating rates shown in Fig. 1 where the SAN had the fastest beating rate at 267.5 ± 13.6 bpm and the significant bradycardia of the SAP (144.1 ± 8.6 bpm) was increased to 214.4 ± 14.4 bpm by TBX18 expression, an improved rate that remained slower than the SAN rate.

## Discussion

### Summary of main results

We screened three novel non‐HCN‐based strategies for biopacemaking in SSS using subsidiary atrial pacemaker tissue from the inferior portion of the sinoatrial node pacemaker complex. NCX1 and TBX3 were unsuccessful in reaching the primary goal of heart rate acceleration, but we report the first proof of concept for the use of TBX18 overexpression as a biopacemaker strategy for SSS. The Ad‐TBX18‐infected SAP displayed a significantly accelerated spontaneous pacing rate, there was improved heart rate stability and heart rate response to isoprenaline was restored. These are features that would be beneficial in treating the clinical syndrome of SSS, which includes bradycardia, sinus pauses and chronotropic incompetence. These effects were primarily mediated by TBX18‐induced upregulation of HCN2 and an overall increase in HCN channel abundance; upregulation of RYR2 was also seen. Biophysically detailed computer modelling suggested that the measured changes in HCN channel abundance could account for the majority of the observed effect on spontaneous pacing rates. Upregulation of RYR2 was also predicted to have a small effect on rate acceleration.

### Effect of TBX18 on gene expression in the SAP

In our experiments expression of TBX18 was an effective biopacemaker strategy; TBX18‐induced enhancement of pacemaker function in the SAP is primarily driven by an increase in the overall abundance of HCN channels, primarily the HCN2 isoform with a contribution from the upregulation of RYR2. Contrary to what might be predicted there was no effect on expression levels of Cx43.

Expression of TBX18 in ventricular myocytes has been demonstrated to reprogram ventricular myocytes to SAN‐like cells with spontaneous pacemaking activity that is driven by upregulation of HCN4 and enhancement of the Ca^2+^ clock (Kapoor *et al*. 2013). Furthermore repression of Cx43 expression has been shown in neonatal rat ventricular myocytes and porcine ventricular myocardium (Kapoor *et al*. 2013; Hu *et al*. 2014). These differences may be due to species‐ or tissue‐specific effects of TBX18. Although one group has demonstrated reprogramming of ventricular myocytes to pacemaker phenotype in rat, guinea pig and porcine ventricular cardiomyocytes (Kapoor *et al*. 2013; Hu *et al*. 2014), others have reported only partial phenotypic remodelling in mouse atrial myocardium (Greulich *et al*. 2016). TBX18 imparts a central SAN phenotype onto TBX3‐positive tissue during fetal cardiac development and TBX18 knockout mice display severe hypoplasia of the central SAN ‘head’, but the SAN ‘tail’ region develops normally (Christoffels *et al*. 2006; Wiese *et al*. 2009). Expression of TBX18 and TBX3 are required for the development of the normal SAN, and the absence of TBX3 from mature cardiomyocytes may hinder the use of TBX18 as a biopacemaker in the working myocardium.

In SAP tissue there is a high level of constitutive TBX3 expression and this may allow TBX18 to impart a more faithful pacemaker program with gene expression that differs from that seen when expressed in working myocardium that has low levels of TBX3 (Morris *et al*. 2013). TBX3 is specifically expressed in the cardiac conduction system even in the mature adult heart (Hoogaars *et al*. 2004), working by suppression of differentiation of nodal tissue to working myocardium, allowing these cells to acquire the pacemaker phenotype (Hoogaars *et al*. 2004). However, TBX3 overexpression alone cannot completely reprogram mature cardiomyocytes into pacemaker cells, and induced overexpression of TBX3 was not sufficient to increase HCN4 expression in adult mouse atria (Bakker *et al*. 2008). Our data demonstrate that expression of TBX3 had no effect on the pacemaker rate of the SAP, perhaps because the TBX3 level is already high in this tissue (Morris *et al*. 2013).

### Biopacemaker effect

The observed effect of HCN2 on the spontaneous pacemaker rate of SAP tissue is in keeping with our previous findings (Morris *et al*. 2013). We have previously demonstrated that upregulation of HCN4 alone in the SAP does not enhance pacemaking, and only HCN2 or HCN212 (a chimaeric HCN channel) was effective (Morris *et al*. 2013). These findings are intriguing given that HCN4 is the primary HCN isoform in the SAN and will therefore contribute the majority of *I*_f_ to SAN pacemaking under normal conditions (Chandler *et al*. 2009). The importance of HCN4 to cardiac pacemaking is underscored by the fact that mutations in the HCN4 gene are known to cause familial bradycardia (Schulze‐Bahr *et al*. 2003; Milanesi *et al*. 2006). However the exact role of HCN4 in setting the resting heart rate and accelerating the heart rate is not entirely clear; many patients with HCN4 mutations have a resting bradycardia but normal heart rate response to exercise (Laish‐Farkash *et al*. 2010; Schweizer *et al*. 2010; Duhme *et al*. 2013), a similar picture is seen an inducible HCN4 knockout mouse though a cardiac‐specific inducible HCN4 knockout mouse did display severe generalised bradycardia (Herrmann *et al*. 2007). The context dependence of HCN channels may limit the activation of HCN4 in tissue outside the central SAN (Qu *et al*. 2002).

The HCN1 and HCN2 isoforms may be more effective because HCN2 has more favourable activation kinetics than HCN4, and HCN1 activates more rapidly than the other isoforms (Biel *et al*. 2002). In addition HCN2 has greater cAMP sensitivity than the other cardiac isoforms, and this may account for the enhanced isoprenaline sensitivity seen in the SAP‐TBX18 preparations; the physiological significance of this would need careful assessment in a large animal model. Biophysically detailed computer modelling presented here is consistent with the concept that increasing the overall HCN abundance, predominantly via HCN2, can increase the pacing rate of nodal tissue without the need to increase levels of HCN4. In further support of this, other biopacemaker groups have similarly focused on these HCN isoforms with more favourable kinetics to achieve pacemaking with physiologically relevant heart rates (Qu *et al*. 2003; Plotnikov *et al*. 2004; Bucchi *et al*. 2006; Tse *et al*. 2006).

Use of TBX18 would have theoretical advantages over the use of HCN2 or HCN212 alone, promising to alter several gene targets simultaneously including multiple HCN isoforms along with Ca^2+^‐based mechanisms, and therefore more faithfully recapitulating the complex nature of the SAN. Our present work supports this assertion by demonstrating HCN2 and RYR2 upregulation, a trend towards HCN1 upregulation, and a change in HCN isoform ratio. TBX18 *versus* single HCN ion channel expression in SAP tissue has not been directly compared and so translation to a large animal model or human use would need systematic assessment of both strategies.

### Pacemaker stability

In addition to a general bradycardia, rate instability is an important feature of clinical SSS in the form of periodic severe bradycardia and pauses. Because our SAP preparations are *ex vivo*, and therefore free of autonomic input, we were able to use measures of HRV to quantify intrinsic pacemaker stability in detail over 2 h, which corresponds to approximately 24,000 beats. TBX18 overexpression significantly improved heart rate stability in the SAP. In keeping with this, prior study of a cardiac‐specific HCN2 knockout mouse found rate instability and sinus pauses, although there was not a resting bradycardia (Ludwig *et al*. 2003). In this study HRV was used to assess heart rate stability in a denervated preparation, but the effects of a possible reduction in HRV in a large animal model would need to be assessed.

When comparing HRV (as a measure of rate stability) in the native SAN *vs*. SAP, we expected higher HRV in SAP tissue due to the likelihood of less stable pacemaking in the SAP tissue. There was a trend for the SAP to demonstrate more instability but this did not reach statistical significance. The study was powered for the primary outcome measure, which was a difference in pacing rate. The HRV in both the SAN and the SAP had a large standard deviation, likely representative of physiological heterogeneity of the animals and the unpredictable nature of pacemaker behaviour. A reduction in the standard deviation of this measure would have required prolonged recordings, but we have previously shown that culture of this tissue beyond 48 h has a high risk of infection and tissue degradation (data not shown).

There was a trend to reduced HRV in the TBX18‐treated SAP compared to the SAN although not reaching statistical significance. The reasons for this are not clear, but it may represent a more homogeneous population of pacemakers, i.e. the biopacemakers induced by TBX18 are more similar to each other than those within the native SAN, which are complex and heterogeneous in ultrastructure. However, we acknowledge that this difference in physiology may be relevant *in vivo* and as such would require further assessment in a large animal model in the future.

### Overdrive suppression

SAN tissue showed low overdrive suppression compared to uninjected SAP tissue but there was no change demonstrated in cRT between SAP and SAP‐TBX18. Overdrive suppression is thought to be related to both Na^+^‐ and Ca^2+^‐based mechanisms (Boyett & Fedida, 1984; Watanabe *et al*. 1996; Boyett MR *et al*. 2000). In one early study on canine Purkinje fibres, short trains of overdrive pacing produced less suppression in low Ca^2+^ solutions and more suppression in high Ca^2+^ solutions, and low Na^+^ solutions also led to a reduction in overdrive suppression (Musso & Vassalle, 1982). The implication was that the Na^+^/Ca^2+^ exchanger NCX might be involved. It also suggested greater suppression was associated with higher intracellular Ca^2+^, increased K^+^ conductance and increased hyperpolarization (Musso & Vassalle, 1982). cRT was affected in another study by ryanodine showing greater overdrive suppression in the context of 0.1 μM ryanodine and a reduction in sarcoplasmic reticular function (Bassani *et al*. 1999). Ad‐Tbx18 infection in our work did not induce any change in Na^+^ channels, K^+^ channels or NCX, which may offer an explanation for the lack of change in overdrive suppression.

### Limitations

The primary limitation of this study is that SSS is a complex disease that is hard to model in animals or *in vitro*. Although SAP tissue in our work faithfully recapitulated bradycardia, which is the primary physiological pathology of SSS along with a degree of heart rate instability, there is evidence that idiopathic SSS in humans may be associated with a wider atrial myopathy that perhaps accounts for the increased incidence of atrial fibrillation in these patients (Sanders *et al*. 2004). We accept that the cause of idiopathic SSS is not completely understood and there may be structural remodelling in and around the SAN that would not be treated by localised TBX18 expression. This would need to be further assessed in large animal models and addressed before any future human trials.

We were unable to measure action potentials directly from the leading pacemaker site for comparison with the modelling data due to fragility of the tissue after culture. We have found that after 48 h in culture, handling or transfer of the tissue to different superfusion solutions frequently results in cessation of pacing activity both in control and virus‐treated preparations. Similarly semi‐quantitative demonstration of changes in ion channel protein using immunohistochemistry was not possible as the technique was not reliable using sections taken from cultured tissue, and therefore we were forced to rely on mRNA level as the only indicator of changes to expression.

### Translation to clinical application

Further steps are required to translate this work to clinical application. As with all research using virally mediated gene expression, the principle hurdle is stable, long‐term gene expression. Demonstration of a physiologically relevant heart rate in a large animal model using TBX18 overexpression may require dosing experiments to achieve the desired effect.

### Summary

We describe the first proof of concept for the use of TBX18 as a biopacemaker strategy for SSS, a common disease that causes significant morbidity and mortality, and the first detailed analysis of the effect of a biopacemaker on rate stability. The primary effects of improved pacing rate, improved rate stability and enhanced adrenergic sensitivity are driven by upregulation of HCN2. This is in keeping with the known electrophysiological properties of the HCN2 channel and the observed phenotype of HCN2‐deficient mice.

## Additional information
